# A prospective surgical evaluation of the coexistence of endometriosis and interstitial cystitis/bladder pain syndrome

**DOI:** 10.1007/s00404-026-08507-y

**Published:** 2026-07-08

**Authors:** Giovanni Favero, Felix Zeppernick, Magdalena Zeppernick, Tatiana Pfiffer, Thilo Schwandner, Ivo Meinhold-Heerlein

**Affiliations:** 1 Department of Gynecology and Obstetrics Justus-Liebig Universität Giessen, University Hospital Giessen-Marburg (UKGM), Campus, Giessen, Germany; 2 Asklepios Hospital Lich, Lich/Justus-Liebig Universität Giessen, Lich, Germany

**Keywords:** IC/BPS, Endometriosis, Association

## Abstract

**Background:**

Endometriosis and interstitial cystitis/bladder pain syndrome (IC/BPS) frequently coexist; however, diagnostic delays and non-standardized criteria limit accurate identification of these conditions.

**Objective:**

To assess the feasibility, safety, and clinical characterization of coexisting endometriosis and IC/BPS using a standardized surgical evaluation approach.

**Methods:**

In this prospective single-center study, approximately 100 women with presumed endometriosis and bladder symptoms undergoing laparoscopy for staging and/or treatment will simultaneously undergo diagnostic cystoscopy for IC/BPS. Optical confirmation and phenotype characterization of IC/BPS will be assessed. In cases with cystoscopic signs of IC/BPS, a standardized therapeutic protocol will be initiated to address bladder-centric symptoms alongside endometriosis treatment.

**Results:**

The study will evaluate whether systematic surgical assessment enables reliable detection of coexisting endometriosis and IC/BPS and facilitates the identification of bladder-centric and non–bladder-centric IC/BPS phenotypes. Early recognition of IC/BPS in women with endometriosis may reduce unnecessary interventions and inform individualized management strategies.

**Discussion:**

The combination of standardized laparoscopy and cystoscopy may improve diagnostic precision in patients with suspected coexisting endometriosis and IC/BPS. The study is expected to provide insights that support phenotype-driven, multidisciplinary care and inform future research and the development of integrated diagnostic algorithms.

## What does this study adds to the clinical work

**Table:** 

This prospective study intends to highlight that Endometriosis and IC/BPS may coexist and contribute to persistent pelvic pain. Combined laparoscopic and cystoscopic assessment can improve diagnosis, phenotype characterization, and support individualized multidisciplinary management while potentially reducing unnecessary repeated interventions.	

## Introduction

Endometriosis is a benign, estrogen-dependent, and heterogeneous gynecological disorder characterized by the presence of endometrial-like tissue outside the uterine cavity, resulting in a persistent inflammatory response [1]. This condition affects approximately 10–15% of women of reproductive age and is associated with a wide spectrum of clinical manifestations [2]. Common symptoms include dysmenorrhea, dyspareunia, and abdominal pain, while some patients may also experience dyschezia, dysuria, and urinary frequency. [3, 4] These symptoms often result in a significant reduction in quality of life. Disease extent and severity are described using several classification systems, most commonly the Revised American Society for Reproductive Medicine (rASRM) score and the #Enzian classification [5, 6]. While the rASRM system provides a general assessment of superficial endometriosis, the #Enzian classification is better suited for staging deep infiltrating and more severe forms of the disease [7].

Recently, phenotype-based classification systems have been proposed, offering a more comprehensive framework by integrating lesion morphology and anatomical involvement [8]. Four principal phenotypes of endometriosis may occur independently or concurrently: superficial peritoneal endometriosis, ovarian endometriosis, uterine endometriosis (adenomyosis), and Deep Endometriosis (DE) [9]. Deep Endometriosis is characterized by nodular lesions infiltrating beneath the peritoneal surface (> 3 mm) or penetrating the muscular layer of pelvic or visceral organs, frequently inducing fibrotic reactions and distortion of normal anatomy [10]. This subtype is considered a severe form of the disease, affecting approximately 20% of women with pelvic endometriosis and often involving pelvic organs extensively, leading to significant morbidity [10].

To address the critical 8-to-10-year diagnostic latency, the current European Society of Human Reproduction and Embryology (ESHRE–2022) and American Society Reproductive Medicine (ASRM—2024) guidelines have moved from a surgery-dependent model to a multimodal clinical framework that integrates structured screening questionnaires, physical exam, and high-resolution imaging (Ultrasonography/Magnet Resonance Imaging) for non-invasive prediction of the disease [11, 12]. This concept intends to facilitate initiation of empirical treatment, while reserving laparoscopy with histological verification (traditionally the definitive gold standard) for complex or imaging-negative cases. More recently, the development and progressive implementation of microRNA signatures, together with the use of targeted questionnaires, has shown promise in expediting diagnosis [13, 14]. Earlier identification enables timely therapeutic intervention, which may not only slow lesion progression but also help prevent pain centralization and chronification. First-line management of endometriosis consists of medical therapy with combined oral contraceptives or progestin-based treatments [12]. In cases of treatment failure, surgical intervention is considered as the second-line therapy, with the extent of surgical radicality tailored to the patient’s symptoms and reproductive desires [15, 16].

At the molecular level, endometriosis progression is driven by dysregulated pathways involved in cellular adhesion, invasion, and resistance to apoptosis [17]. Elevated local estradiol production and progesterone resistance promote lesion survival and proliferation [18, 19]. Inflammation and immune dysfunction are central to endometriosis pathogenesis. Activated peritoneal macrophages secrete excessive proinflammatory cytokines, while reduced natural killer cell activity impairs immune-mediated clearance of ectopic endometrial cells [20, 21]. Moreover, recurrent microbleeding from the lesions contributes to tissue damage, perpetuating chronic inflammation, fibrosis, and pain [22]. Consequently, endometriosis is associated with chronic pelvic pain (CPP) in up to 80% of affected women [23].

Chronic pelvic pain (CPP) is a common and debilitating condition affecting approximately 6–25% of women of all ages worldwide, with reported prevalence varying depending on the definition applied [24, 25]. To date, no universally accepted definition or diagnostic criterion for CPP has been established. The American College of Obstetricians and Gynecologists (ACOG), in collaboration with the ReVITAlize initiative, defines chronic pelvic pain (CPP) as persistent non-cyclic lower abdominal pain lasting at least 6 months. The pain may be constant or intermittent and can be exacerbated by menstruation or sexual intercourse [26]. CPP is associated with significant impairments in quality of life, reduced work productivity, and increased healthcare utilization [25]. A recent systematic review estimated the annual economic costs of CPP to be approximately USD 2.8 billion [27]. From a clinical perspective, CPP accounts for nearly 10% of gynecological consultations, approximately 12% of hysterectomies, and more than 40% of diagnostic laparoscopies [26, 28].

Although the pain is localized to the pelvic region, its perception is ultimately mediated by central nervous system (CNS) processing [29]. Endometriosis shares multiple clinical and pathophysiological features with chronic pelvic pain. Women with these conditions often exhibit alterations in brain structure and function, consistent with central sensitization and dysfunctional pain processing mechanisms. In endometriosis-associated pain, persistent peripheral inflammation and nociceptive input can further drive central sensitization, contributing to pain persistence even after lesion resection or hormonal suppression. Therefore, early and accurate diagnosis, followed by timely initiation of therapy, is critical to interrupt these processes and potentially prevent pain chronification and long-term CNS alterations [30]. Nevertheless, delayed diagnosis remains a major challenge in the management of both CPP and endometriosis, with up to 50% of affected women remaining without a definitive diagnosis even after years of symptoms [31]. Despite its high prevalence and substantial socioeconomic impact, CPP has been described by the World Health Organization (WHO) as a “neglected reproductive health morbidity” [25]. This designation reflects persistent deficiencies in healthcare systems, prioritization, and resource allocation across different regions of the world [31].

In the context of chronic pelvic pain (CPP), the presence of a prior diagnosis of endometriosis should not preclude comprehensive evaluation for additional or overlapping etiologies. The differential diagnosis spans multiple medical specialties, including gastrointestinal, gynecologic, urologic, musculoskeletal, and psychiatric or psychosomatic disorders [32] Management of CPP is extremely challenging due to the wide range of nonspecific symptoms and numerous possible underlying conditions, including depression—affecting up to 50% of patients—and anxiety [25]. Despite the frequent use of invasive diagnostic procedures, patients with CPP are often treated empirically for presumed diagnoses, which frequently results in poor response [31]. As a result of their frustration with these suboptimal outcomes, many patients consult multiple healthcare providers and undergo repeated medical and surgical interventions [32] .

Pelvic pain of bladder origin has received increasing recognition since the initial descriptions of interstitial cystitis (IC) in 1887 and painful bladder syndrome (PBS) in 1957 [33]. These conditions are defined by the American Urological Association (AUA) and the International Continence Society (ICS) as chronic disorders characterized by pain, pressure, or discomfort perceived to originate from the urinary bladder, accompanied by lower urinary tract symptoms, such as dysuria, nocturia, frequency, and urgency, persisting for more than 6 weeks in the absence of infection or another identifiable cause [34, 35]. In contrast, the European Society for the Study of Interstitial Cystitis defines the condition based on the presence of at least one urinary symptom in combination with characteristic cystoscopic findings [36]. Although it was once considered a rare condition, more recent studies indicate a prevalence of 2.7–6.5% among women, with peak incidence occurring between 40 and 60 years of age [33, 34]. While interstitial cystitis (IC) traditionally refers to cases with characteristic bladder findings, such as Hunner’s lesions identified on cystoscopy, bladder pain syndrome (BPS) represents a broader, symptom-based diagnosis that includes patients experiencing bladder-associated pain in the absence of these specific cystoscopic features [37].

**Fig. 1:**
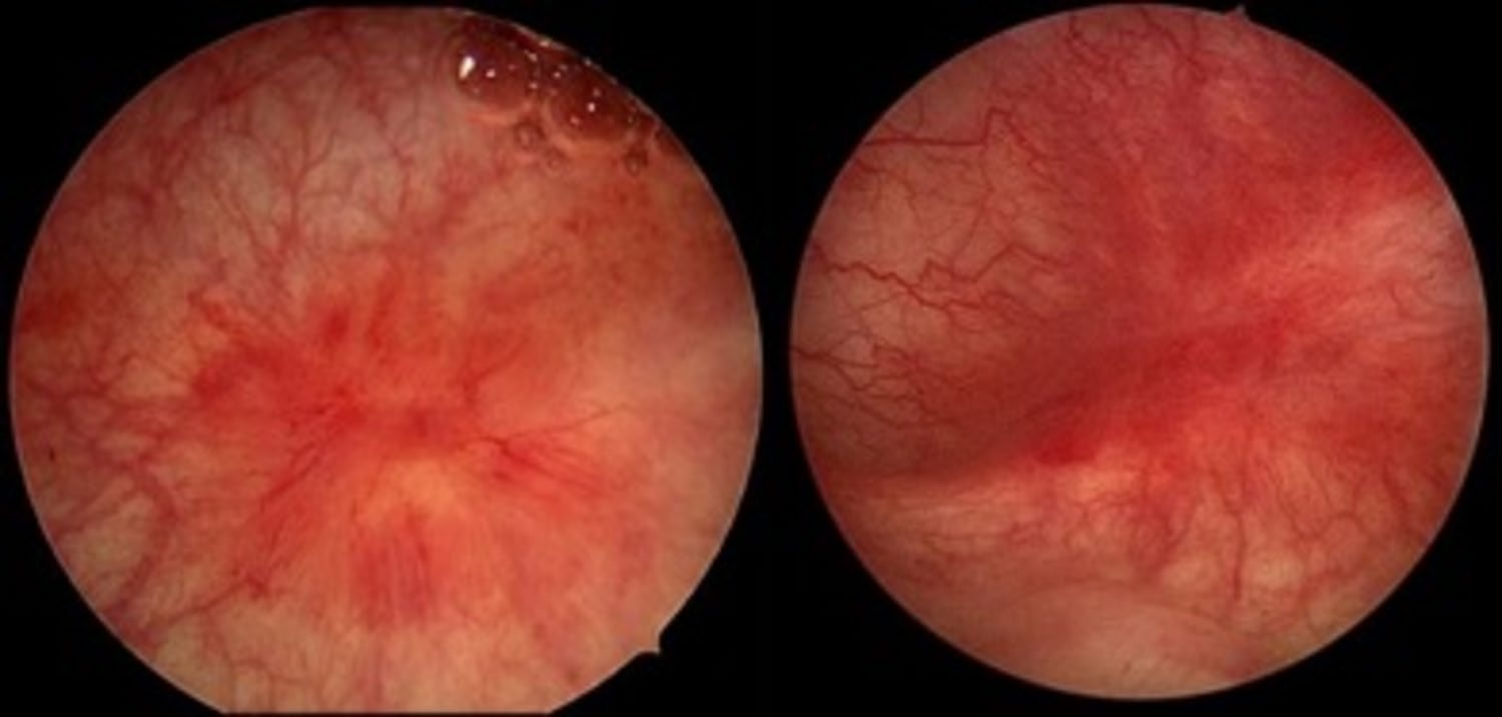
Hunner lesions—severe radiated inflammatory area

**Fig. 2:**
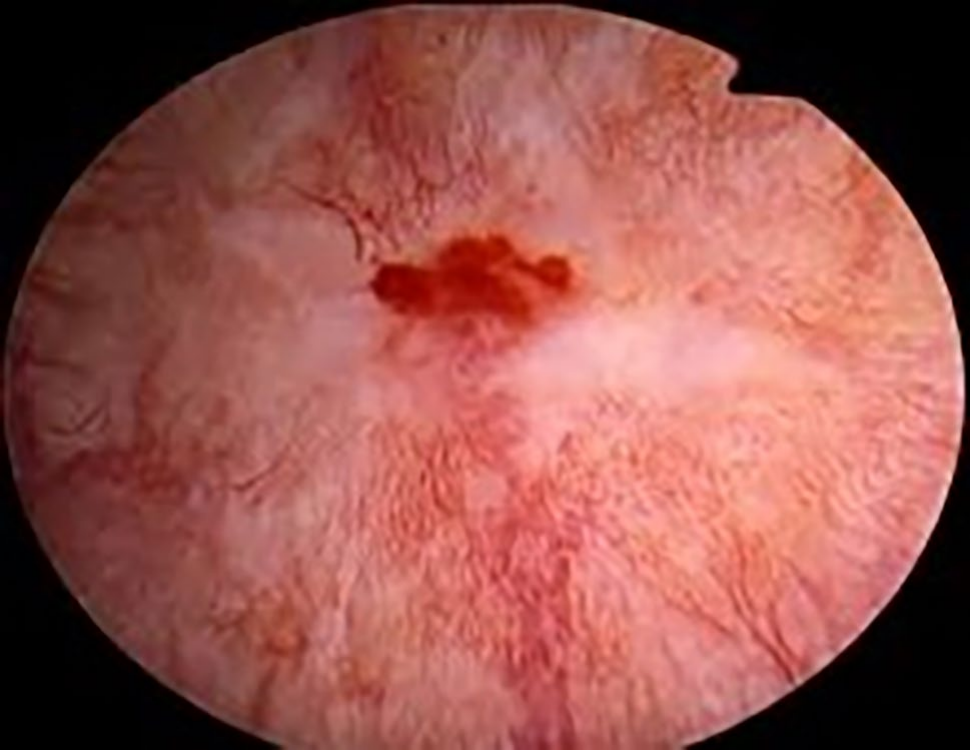
Hunner lesion—small vessels radiating toward a central scar

**Fig. 3:**
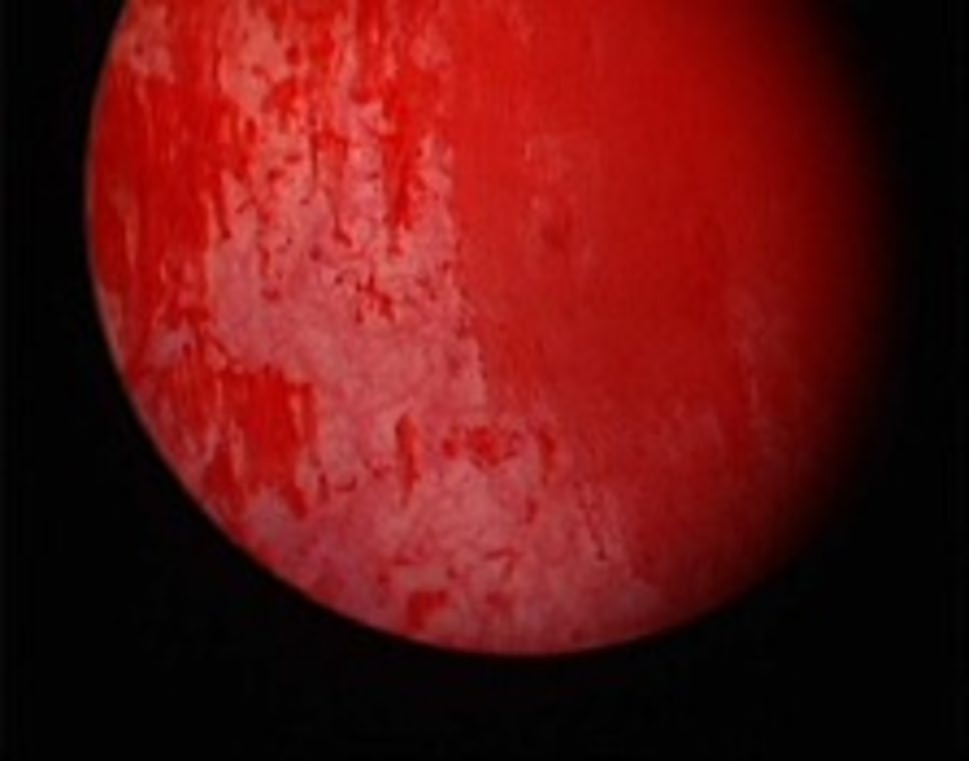
Glomerulations—petechial hemorrhages after hydrodistention

**Fig. 4:**
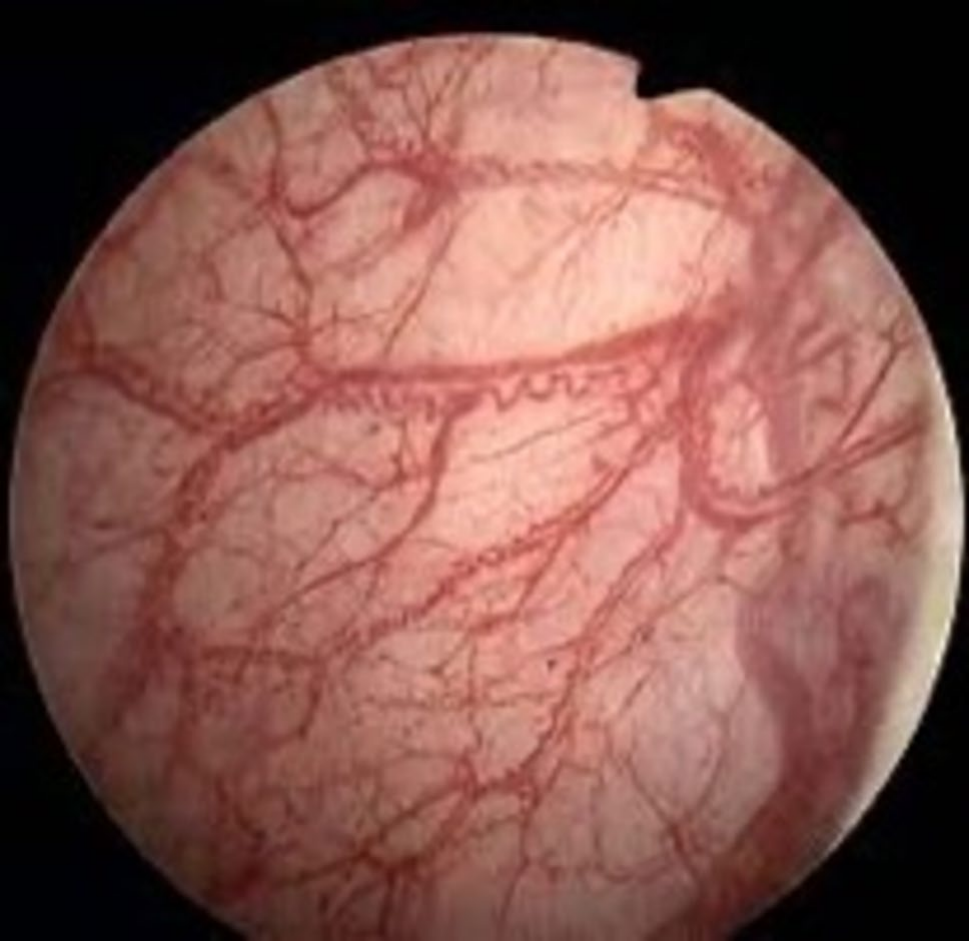
Increased vascularity, and prominent and spiraled blood vessels

Cystoscopy plays a central role in the diagnosis of IC/BPS, enhancing diagnostic confidence through direct visualization of characteristic bladder findings. The most commonly observed cystoscopic features include Hunner’s lesions, glomerulations, and areas of mucosal erythema or edema (Figs. 1, 2, 3, 4). Increasing evidence indicates that IC/BPS is a heterogeneous condition, encompassing distinct subtypes that differ in underlying pathophysiology, clinical presentation, and response to treatment. On the basis of cystoscopic findings, two IC/BPS sub-phenotypes have been described.

- Patients with Hunner’s lesions are classified as having a bladder-centric IC/BPS phenotype, accounting for approximately 20% of cases. This subtype is characterized by chronic inflammatory changes, including mast cell and lymphocyte infiltration, and typically presents with focal bladder pain and visible mucosal lesions on cystoscopy. Patients often demonstrate significant symptom improvement following lesion-directed therapies, such as fulguration, resection, or intralesional steroid injection.
- Patients without Hunner’s lesions are classified as having a non–bladder-centric IC/BPS phenotype, accounting for approximately 80% of cases. This subtype is more commonly associated with systemic pain disorders, including fibromyalgia, endometriosis, depression, irritable bowel syndrome, vulvodynia, dyspareunia, and migraine. It is thought to involve heightened nociception and central sensitization rather than localized bladder inflammation. Multimodal pain management is essential. Treatment options include oral pharmacotherapy—such as pentosan polysulfate sodium, hydroxyzine, amitriptyline, and pregabalin, with or without adjunctive intravesical therapies, including hyaluronic acid. Particular emphasis is placed on neuromodulatory approaches (e.g., antidepressants and antiepileptics), as well as non-pharmacological interventions such as transcutaneous electrical nerve stimulation (TENS), meditation, and structured physiotherapy with pelvic floor rehabilitation.

Notably, Chung et al. (2002) were among the first investigators to describe the association between interstitial cystitis/bladder pain syndrome (IC/BPS) and endometriosis [38]. This observation led Chung to coin the term *“evil twin syndrome”* to denote the coexistence of these two conditions. These patients are significantly more likely to carry a nonbladder-centric IC/BPS phenotype as well as several comorbid, systemic pain diagnoses [39]. Evidence from the literature suggests that this association is particularly pronounced in women with chronic pelvic pain. Reported prevalence rates of coexistence range from 15.5 to 78.3%, reflecting substantial heterogeneity in the individual prevalence of each condition across studies [31]. This variability is largely attributable to differences in diagnostic criteria, the absence of standardized definitions for IC/BPS, and selection biases inherent in study populations. Similarly, the true prevalence of endometriosis remains uncertain due to underdiagnosis, diagnostic delays, and variability in surgical and histopathological assessment. Consequently, precise epidemiological data regarding the coexistence of these conditions are still lacking.

Similar to endometriosis, IC/PBS is frequently associated with a substantial diagnostic delay [39]. Driscoll et al. reported a median symptom duration of 5 years prior to diagnosis, with most patients initially presenting with a single symptom and gradually developing the full clinical spectrum [40]. Delayed recognition often leads to serial interventions, and these patients are more frequently subjected to surgical procedures, including hysterectomy, with limited benefit for persistent pain. It is more likely that many women undergoing repeat surgery for endometriosis refractory to medical therapy already have unrecognized IC/PBS [31]. Thus, despite being a diagnosis of exclusion, the presence of endometriosis does not preclude concomitant IC/PBS.

In this context, we developed a prospective clinical study utilizing standardized diagnostic criteria for both endometriosis and interstitial cystitis/bladder pain syndrome to enhance diagnostic accuracy, inform individualized management, and elucidate the association between IC/BPS and specific endometriosis phenotypes and stages.

## Patients and methods

### - Study design

This is a single-center (Endometriosis Center-University Hospital Giessen-UKGM, Germany), prospective, open-label, single-arm, prospective exploratory interventional feasibility study.

All eligible participants will undergo the specified interventions.

### – Participants

Inclusion criteria. Participants are eligible if they meet all the following.

- positive validated Endometriosis Questionnaire (Zeppernick et al, 2025) [13];
- Indication for Endometriosis surgery, based on fertility, medical therapy resistance, or radiology;
- positive validated Cystitis Questionnaire (O’Leary-Sant et al, 1997) [41];
- Pre-menopausal status;
- Age 18 and 50 years at enrollment;
- Written informed consent after a thorough explanation of study procedures, potential risks, and benefits.

Exclusion criteria. Participants are excluded if any of the following apply:

- Age < 18 or > 50 years at enrollment;
- Significantly compromise ability to complete the study protocol;
- Pregnancy, lactation, or possibility of pregnancy;
- Negative Cystitis Questionnaire (O’Leary-Sant et al., 1997) [41];
- Presence of suspicious lesions during cystoscopy.

Imaging assessments will routinely include ultrasonography, and in cases of clinical suspicion of deep endometriosis, pelvic contrast-enhanced MRI endometriosis findings will be evaluated by one radiologist and a specialized group of gynecologists. Deep Endometriosis will be classified according to #Enzian scores [6] .

### Interventions

The study protocol will encompass the following phases (Chart Flow):
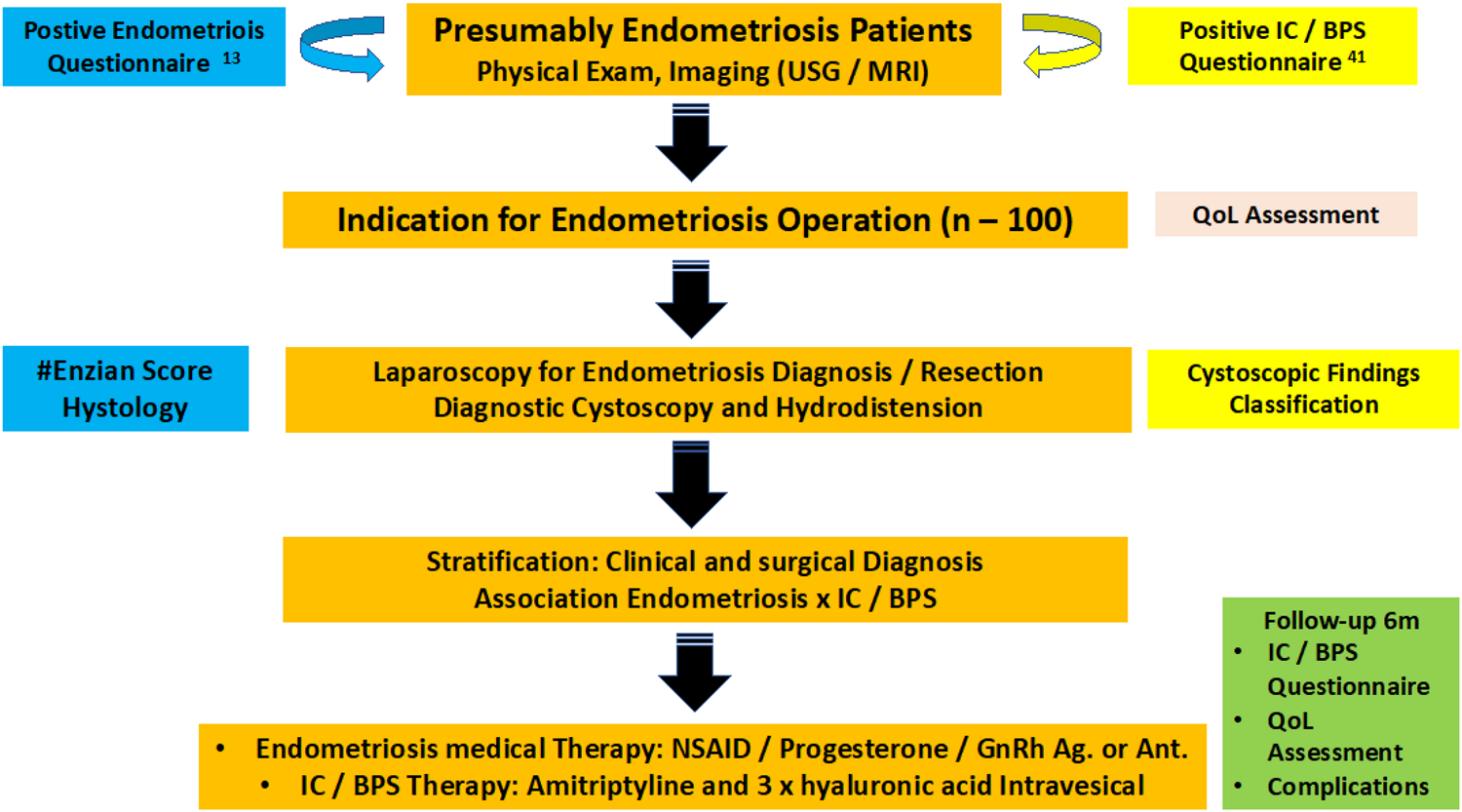

- **Intervention 1:** Operative laparoscopy for endometriosis excision and staging according to #Enzian classification [6].
- **Intervention 2:** Diagnostic cystoscopy simultaneously to laparoscopy. Optical evaluation of IC/BPS and stratification in 2 different phenotypes—bladder-centric and nonbladder-centric. Targeted bladder biopsies will be performed only in the presence of suspicious lesions and not systematically**.** IC/BPS was defined by the presence of a positive, validated Cystitis Questionnaire (O’Leary-Sant et al., 1997) and at least one typical cystoscopic criterion, in accordance with the recommendations of the European Society for the Study of Interstitial Cystitis. [36, 41] .

Cystoscopic findings are as follows:

1. Hunner lesions—Figs. 1, 2: They are not actually ulcers but rather distinct inflammatory areas:
2. Appearance: Circumscript, reddened mucosal areas with small vessels radiating toward a central scar.
3. Behavior: They may crack and bleed (the "cascade" effect) as the bladder fills during hydrodistention.
4. Significance: Patients with Hunner lesions often report higher pain levels and more severe urinary frequency.
5. Glomerulations (petechial hemorrhages)—Fig. 3: They are tiny, pinpoint bleeds in the bladder mucosa that appear after the bladder is distended and then emptied.
6. Structural & vascular changes—Fig. 4: Beyond specific lesions, the cystoscopic exam looks for general signs of chronic inflammation:
7. Increased vascularity: Prominent or friable blood vessels on the bladder wall.
8. Mucosal friability: The bladder lining may appear fragile and bleed easily upon contact or distention.
9. **Intervention 3:** Patients who meet the clinical and cystoscopic criteria for nonbladder-centric IC/BPS will receive adjuvant pain management consisting of amitriptyline (75 mg/day) for 6 months and three sessions of intravesical hyaluronic acid instillations. In addition, patients undergo appropriate medical therapy for endometriosis (progesterone, NSAIDs, GnRH antagonist, or GnRH agonist) based on disease stage (#Enzian classification), fertility desire, and surgical findings.

### Quality of life and toxicity assessment

Patient-reported outcomes will be assessed at baseline and prespecified intervals using validated quality-of-life instruments, including the Endometriosis Health Profile (EHP-30), and the O’Leary-Sant Interstitial Cystitis Symptom and Problem Indices (ICSI/ICPI). Finally, all adverse events and complications will be systematically graded according to the Clavien–Dindo classification and continuously reported.

### Follow-up and outcome assessment

Post-treatment surveillance will be standardized at the University Hospital Giessen-UKGM, Germany to evaluate endometriosis control and functional outcomes through clinical and sonography exams six months after therapy, with recurrence defined as the date of symptom return.

### Sample size

This single-arm Phase II study will enroll a target sample of 100 patients. The sample size is intended to generate preliminary data on feasibility, efficacy, and safety to inform the design of a future Phase III trial. An interim analysis will be conducted after enrollment and primary outcome assessment of the first ten patients. In the case that clinically relevant complications related to cystoscopy or IC/BPS-directed therapy are observed at interim analysis, patient enrollment will be suspended and the feasibility of the study will be reassessed.

### Statistical analysis

The full analysis set will include all participants who initiate study treatment and have at least one post-baseline assessment. The safety population will comprise all participants who receive all three of the study interventions. Data will be recorded in a secure, non-networked database. Descriptive statistics will be used to summarize demographic variables, baseline characteristics, and study outcomes.

The primary endpoint, rate of association endometriosis and IC/BPS, will be estimated along with its corresponding confidence interval. Secondary endpoints, including improvement in symptoms and quality of life (QoL) after individualized management, and elucidating the association between IC/BPS and specific endometriosis phenotypes and stages will be analyzed using the Kaplan–Meier method. Adverse events will be summarized by type, severity grade, and frequency.

Missing data for patient-reported outcomes will be managed using the last observation carried forward approach. Given the exploratory nature of this study, a formal sample size calculation was not performed. The selected sample size of 100 participants reflects the approximate frequency of bladder-centered symptoms among endometriosis patients at a single center, as well as ethical considerations to minimize exposure to medical and operative interventions prior to completion of the planned interim safety analysis. If sufficient events occur, exploratory multivariable analyses using Cox proportional hazards regression will be conducted.

### Timeline

The study will commence following approval by the Certified Clinical Research Review Board and is scheduled to conclude in approximately 2 years.

### Ethical considerations

This study will be conducted in accordance with the principles of the Declaration of Helsinki, the Ethical Guidelines for Medical Research Involving Human Subjects in Germany, and all applicable national and international regulations. Ethical approval was obtained from the Certified Clinical Research Review Board of University of Giessen. Written informed consent will be obtained from all participants prior to study enrollment. During the informed consent process, participants will be clearly informed that the treatment strategy is non-standard and exploratory in nature and involves small potential risks related to a special approach.

## Results and discussion

This study was designed to investigate the coexistence of endometriosis and interstitial cystitis/bladder pain syndrome (IC/BPS), with a particular focus on diagnostic feasibility, safety, and clinical characterization using standardized surgical criteria. Given the well-documented diagnostic delays associated with both conditions and their potential role in pain chronification and central sensitization, early and accurate identification is increasingly recognized as a critical component of effective management within a multidisciplinary care framework.

Previous studies have suggested a strong association between endometriosis and IC/BPS—often referred to as the “evil twin syndrome”—yet the available evidence is limited, largely retrospective, and derived from small, heterogeneous case series employing variable and frequently non-standardized diagnostic criteria.38 This methodological variability has substantially limited the accurate estimation of their coexistence and hindered the development of targeted diagnostic and therapeutic strategies. In particular, differences in IC/BPS diagnostic definitions and reliance on symptom-based criteria have contributed to wide discrepancies in reported prevalence rates.

The present study is designed to address these limitations by adopting a prospective design and applying well-defined surgical diagnostic criteria for both conditions. Laparoscopy will allow direct visualization and histological confirmation of endometriosis, while concomitant cystoscopy will enable objective assessment of IC/BPS phenotypes, including bladder-centric and non–bladder-centric forms. This integrated approach aims to improve diagnostic accuracy and reduce misclassification, thereby providing a more reliable estimate of disease coexistence.

Importantly, the study prioritizes patient safety by avoiding systematic bladder biopsies and limiting tissue sampling to cases with suspicious cystoscopic findings. This approach balances diagnostic rigor with minimization of procedural risk. Moreover, the study design acknowledges that pelvic surgery itself may influence bladder physiology; therefore, careful interpretation of findings will be required to distinguish pre-existing IC/BPS from procedure-related changes.

Given that women with CPP are frequently exposed to repeated medical and surgical interventions, often without adequate symptom resolution, the identification of coexisting IC/BPS has significant clinical implications. Failure to recognize IC/BPS in patients diagnosed with endometriosis may contribute to persistent postoperative pain and unnecessary surgical escalation. Thus, demonstrating the feasibility and clinical value of systematic cystoscopic evaluation in this population could support a shift toward more comprehensive, phenotype-driven management strategies.

Some limitations of this study should be acknowledged. The single-center design and exploratory nature may limit generalizability, and the absence of universally accepted diagnostic criteria for IC/BPS remains an inherent challenge. Nevertheless, these limitations are acceptable in the context of a feasibility study intended to generate high-quality preliminary data and inform the design of future multicenter trials.

In conclusion, this study aims to provide robust prospective evidence regarding the coexistence of endometriosis and IC/BPS using standardized surgical assessment. By improving diagnostic precision and highlighting clinically relevant IC/BPS phenotypes, the study is expected to generate findings that may contribute to earlier recognition, more individualized treatment strategies, and ultimately better pain-related outcomes. More broadly, this work may support the development of integrated diagnostic algorithms and multidisciplinary care models for women with chronic pelvic pain, with important implications for clinical practice and future guideline development.

## Data Availability

No datasets were generated or analyzed during the current study.
